# Primary-school-aged children inspire their peers and families to eat more vegetables in the *KiiDSAY* project: a qualitative descriptive study

**DOI:** 10.1186/s12887-024-04643-z

**Published:** 2024-03-09

**Authors:** Karpouzis F., Walsh A., Shah S., Ball K., Lindberg R.

**Affiliations:** 1 Institute for Physical Activity and Nutrition, School of Exercise and Nutrition Sciences, Deakin University, Melbourne, VIC 3125 Australia; 2 School of Behavioural and Health Sciences, Australian Catholic University, VIC, Melbourne, Australia; 3 Faculty of Medicine and Health, University of Sydney, Sydney, NSW Australia

**Keywords:** School, Children, Nutrition education, Vegetable consumption, Qualitative research, Focus groups

## Abstract

**Background:**

While vegetable intakes in Australia remain sub-optimal across all age groups, children are rarely consulted about their ideas on how to increase consumption. Qualitative research involving children provides an opportunity to consider their views. The aim of the *Kids initiative inspires Dietary Success in Adults and Youth (KiiDSAY)* project was to explore the views of school-aged children, who had participated in a school-based nutrition education program, about inspiring their peers and families to eat more vegetables.

**Methods:**

A total of 26 children (15 boys) aged 10–12 years from four primary schools in New South Wales, Australia, participated in seven focus group interviews. Purposeful sampling was used to recruit participants. The study involved open-ended semi-structured questions conducted via Zoom that were audio-recorded, transcribed verbatim and analysed using thematic analysis with deductive and inductive coding in NVivo.

**Results:**

Four major themes emerged: (i) taste; (ii) family environment; (iii) healthy eating; and (iv) change makers; with subthemes that were embedded within Social Cognitive Theory and Ecological Model of Health Behaviour theoretical frameworks.

**Conclusions:**

Children’s inputs hold great potential for informing future interventions, particularly when designing or refining school-based nutrition programs. Children offered suggestions on how to inspire increased vegetable consumption among their peers and families that could be taken into consideration for future research and practice. These included: cooking activities in the home and school settings using recipes that creatively hide/mask/enhance the flavour of vegetables, involving positive role models and supportive school environments. Additionally, children recommended a sequential approach to the delivery of recipes starting from fruit-based and transitioning to vegetable-based recipes. Given the challenges faced in increasing children’s vegetable consumption, particular focus on future research in this area is warranted.

**Trial registration:**

FEAST Trial registered 14th December 2020 with the Australian and New Zealand Clinical Trials Registry (ACTRN12620001347954).

**Supplementary Information:**

The online version contains supplementary material available at 10.1186/s12887-024-04643-z.

## Background

Despite decades of behavioural interventions attempting to increase the consumption of fruits and vegetables (F&V), population-level intakes remain suboptimal [1, 2]. The combined daily intake of F&V, should be five portions (i.e. 400 g) for adults [3, 4] and for children, intakes should range between 1–2 cups of fruit and 1–3 cups of vegetables [5]. In Australia, much like other parts of the world [5–9], recommendations are not being met. For example, only 8.7% of adults and 9.0% of children (aged 2–17 years) in Australia, are eating the recommended five servings of vegetables/day [10].

As F&V consumption decreases between childhood and adolescence [11–13] and eating habits track into adulthood [14], much emphasis has been placed on improving dietary habits at a young age [15]. Schools are a popular setting to implement interventions promoting F&V because of their wide and diverse reach [16–18]. Systematic reviews evaluating the impact of school-based programs on F&V consumption have reported mixed results, generally showing low to moderate increases in F&V intakes [19–24]. Most of these modest effects have been attributed to increases in fruit, rather than vegetable consumption [22, 25, 26].

It has been suggested that interventions targeting F&V intakes should be conducted separately [26, 27]. A recent systematic review found evidence that experiential primary-school nutrition programs were more likely to succeed if they targeted vegetable intakes alone [28]. Consistent with this review, an Australian study involving 1403 parents/carers of primary-school-aged children concluded that health promotion may need to be targeted towards vegetable consumption in preference to fruit consumption [29]. Furthermore, a review synthesising qualitative research on children’s views of school-based programs promoting the health benefits of F&V suggested that these food groups should be promoted in different ways [30].

Although fruit has been reported to be well-liked by children, vegetables are less accepted [26, 31, 32]. Dislike for the taste of vegetables commonly emerges as a barrier to their consumption among adults [33, 34] and children [26, 31, 32]. Qualitative enquiries report children describing vegetables as unappealing and tasting bitter [35], their least favorite food [32] and a food to be consumed by ‘grown-ups’ only [36]. Very few qualitative studies have explored how children and adolescents would motivate their peers to eat vegetables [37–39], with no studies identified on how they would motivate their families.

Qualitative research provides opportunities to explore new ideas by giving children an opportunity to voice their views [40]. As such, a qualitative study was designed called *Kids initiative inspires Dietary Success in Adults and Youth* (KiiDSAY). The aim of the KiiDSAY project was to explore children’s perspectives of how they would inspire vegetable consumption within their microsystem i.e. among peers and families, in the home and school environments, after they had participated in the *Food Education and Sustainability Training* (FEAST) program. The FEAST program was created by OzHarvest, an Australian not-for-profit food rescue organization [41]. The program’s purpose was to educate primary-school children about nutrition, food waste and sustainability, while teaching them to cook. It was designed using the PRECEDE-PROCEED Planning model [42] and Social Cognitive Theory (SCT) [43]. A detailed description of the FEAST program has been previously published [44]. FEAST participants were chosen to gain insights about the program, as well as using the opportunity to explore their ideas about vegetable consumption. This article focuses on the latter.

## Methods

The overarching methodology for this study was qualitative description (QD), which is often used to provide answers to questions of relevance to practitioners and policy makers [45]. It offers an opportunity to collect descriptions about phenomena of which little is known, often includes overtones of other qualitative methods and is used as part of mixed methods research [46]. The *Consolidated Criteria for Reporting Qualitative Research* (COREQ) checklist [47] informed this study’s design and reporting. Ethical approval was granted by the Human Ethics Advisory Group, Faculty of Health, at Deakin University in Melbourne, Australia (HEAG-H 151_2020) and New South Wales (NSW) State Education Research Applications Process (SERAP) approval was granted (SERAP #  2019163).

### Research questions

The following research questions were used to guide this project:

- What are the potential facilitators and barriers to eating vegetables from a child’s perspective?
- What are children’s views, opinions and perspectives on how they would inspire their peers and families to eat more vegetables?
- What actions would children take to promote vegetable consumption in their homes and schools?
- What did children like the most about, and how would they improve, the FEAST program?

### Theoretical position

Qualitative description design was chosen as it provides direct rich descriptions of phenomena under investigation [45], without deviating far from the data as the researcher stays close to the “*surface of the data*” but allows for some interpretation [46]. This is consistent with naturalistic research, in that reality is considered subjective and varies from person to person, where participants and phenomena are studied in their natural context [46].

### Theoretical frameworks

**Fig. 1:**
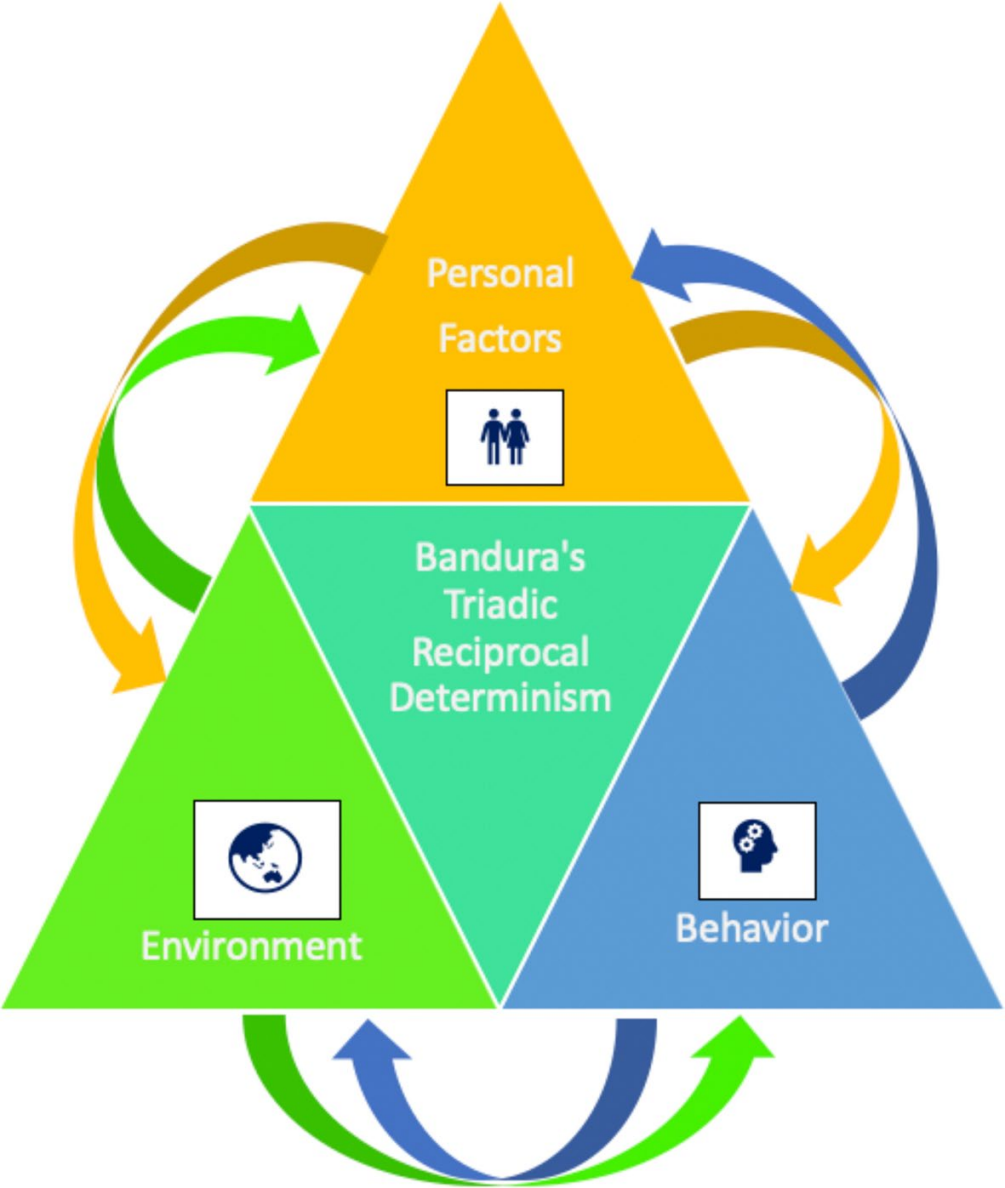
Triadic reciprocal determinism diagram. Legend: Bandura’s Triadic Reciprocal Determinism [48]—two-way influence between personal factors, behaviour and environment; Personal Factors: the cognitive, biological and other internal events that can affect perceptions and actions; Environment: the external environmental factors; Behaviour: What people think, believe, and feel, affects how they behave

This study drew on SCT [48] and the Ecological Model of Health Behaviour (EMHB) [49]. Bandura’s SCT [48] is one of the most widely used behaviourally based theories for the development of nutrition education [50, 51]. This theory underscores the importance of the reciprocal interactions and influences between the personal, behavioural and environmental factors that affect human behaviour [43] (See Fig. 1). The EMHB also recognises the interrelationship between people and their environments [49]. Reciprocal determinism is a core construct that appears in both SCT [43] and EMHB [49]. According to SCT, one of the ways in which children learn is through observation and as such, they are influenced by their peers and adults [43]. Similarly, according to EMHB, children are influenced by their peers and family, at the microsystem level, of which the home and school are their most proximal environments [49]. Conversely, children can also bring about a change in their environment [48]. As such, reciprocal determinism was used from SCT and EMHB along with several other constructs from SCT (i.e. facilitators, barriers, role models and strategies), to create the interview guide and to answer the research questions. Additional file 1: outlines the theoretical frameworks that underpin interview questions with their key constructs.

### Setting, participants and recruitment

Schools that had implemented the FEAST program between 2020–2021 were invited to participate in the KiiDSAY project. Teachers were emailed letters of invitation for distribution to their student’s parents/carers that sought permission for their child to take part in the focus group discussions. Teachers were asked to recruit a mix of male and female students from Grades 5–6 (aged 10–12 years) for focus group interviews during Term 4, 2021. Interviews were scheduled via the Zoom platform in the classroom setting, using the teacher’s computer in the teacher’s presence. Students participating in this study were compensated with a $20.00 AUD supermarket gift voucher and a certificate of appreciation for their time. Additionally, schools received a $100.00 AUD supermarket gift voucher.

### Sampling and sample size

Participant selection was based on purposeful [45, 52] and convenience sampling [46] i.e. students who had participated in the FEAST program (prior to COVID19 school closures). According to Bradshaw et al. 2017 [46], a good *“rule of the thumb is to conduct three or four focus groups”* involving children [53], with 6–8 students per group. These numbers are acceptable for focus group interviews with children [53]. As such, preliminary sample size was set at approximately 24–32 participants, involving four schools. Focus groups provide researchers with large amounts of data on a specific topic in a relatively short period and are appropriate to use among children aged 10–12 years [53].

### Data collection methods

Open-ended, semi-structured questions, which are used in qualitative description studies [46, 54], were used for this study. Data collection involved focus group interviews which were conducted by the lead researcher (FK), and audio recorded on the Zoom platform. The interview questions are in Additional file 1. The interview script has been outlined in Additional file 2. The questions were pilot tested on one 12-year-old girl (the daughter of a schoolteacher and friend of the lead researcher). Notes were taken during interviews and a reflexive journal maintained throughout data collection and analysis.

Parents/carers were provided with information about the study and consent forms to sign in advance. Before interviews commenced, the interviewer provided a brief outline of the study and informed participants of their rights, as well as advising them that they were free to say ‘pass’ anytime they did not feel like answering a question. To limit social desirability bias among participants, children were assured that there were no ‘right’ or ‘wrong’ answers. Participants were asked to provide verbal assent prior to commencement of the focus group interviews. School and participant names were coded to ensure anonymity, for example: ‘*School 2, FG 2, Girl 3’* corresponded to participant three, from focus group 2, from school 2.

### Data analysis

Data analysis was approached systematically, following four key stages: data immersion, coding, development of subthemes, and the identification of major themes [55]. The process of analysis began with data immersion, whereby the lead researcher (FK) listened to, and transcribed the content verbatim. Following transcription, data were imported into QSR-NVivo 12 software [56] for data management and analysis.

Given that SCT and EMHB were used to design the interview questions, the approach to data analysis was deductive. This ‘theory-driven’ approach [57] was used and the following codes were applied: facilitators, barriers, strategies/actions, role modelling, and reciprocal determinism. Along with these theoretical frameworks, themes were able to evolve inductively. As such, a combination of both approaches was used [57, 58]. As thematic analysis is the most commonly used method of analysis in public health research [55, 59, 60], and it can be applied to QD [45, 46, 54], it was used to generate codes from the data [61]. Coding involved assigning descriptive labels to all participant responses [30] using both the deductive and inductive approaches.

**Fig. 2:**
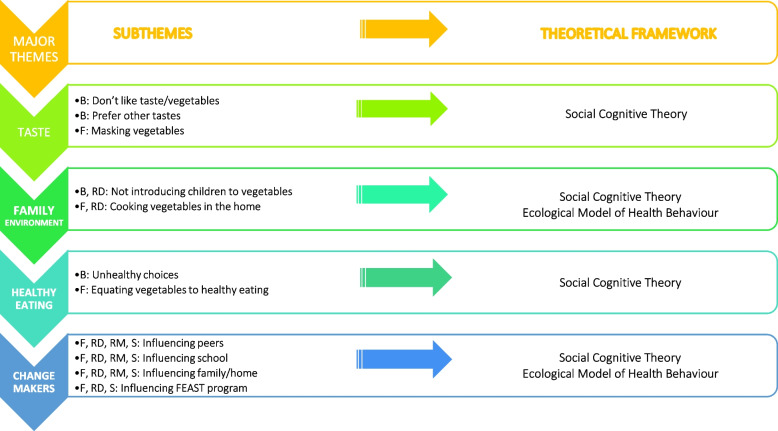
KiiDSAY Project: Major themes and subthemes with corresponding theoretical constructs. Legend: Social Cognitive Theory [B Barriers; F Facilitators; RD Reciprocal Determinism; RM Role Models; S Strategies]; Ecological Model of Health Behaviour [RD Reciprocal Determinism]

Three rounds of coding were undertaken by FK, followed by the grouping of common ideas into subthemes. Two co-authors, RL and AW, independently checked four transcripts, to cross-check coding and emerging subthemes and themes. FK is a PhD candidate and lead researcher evaluating the FEAST program, with experience in interviewing children (one-on-one) in the clinical setting, with some experience conducting focus-group interviews among children aged 5–12 years. RL is a Postdoctoral Research Fellow in public health nutrition and food security and has experience in qualitative research including thematic analysis and interviews. AW is an Accredited Practising Dietitian and National Course Coordinator of the Master of Dietetic Practices, with extensive experience in qualitative research. Discussions between FK, RL and AW followed to refine and/or elaborate on codes, emerging subthemes and themes. The final stage involved identification of major themes that emerged from the deductive and inductive processes. Discussions between authors (FK, RL, AW, SS and KB) were held and consensus was reached, confirming major themes. These discussions helped establish trustworthiness in the findings and analysis. Identification of major themes and how they relate to key constructs of SCT and EMHB has been outlined in Fig. 2.

## Results

Of the thirty-four schools that were invited, five schools agreed to participate and of those, four schools provided informed consent, with one school withdrawing due to the teacher taking leave. Twenty-eight students from Years 4—6 provided opt-in informed consent to participate from their parents/carers and of those, 26 participated, as two students from different schools were absent on the day of the scheduled interviews. Seven focus group interviews were conducted between 19th November and 13th December 2021, with a duration between 30–50 minutes (mean 45 ± 7 min), and involved between 2–6 participants per interview. Table 1 outlines student, teacher and school characteristics.

**Table 1:** Student, teacher and school characteristics for the KiiDSAY Project

	School 1	School 2	School 3	School 4
**School characteristics**				
** Type of school**	Government (Public School)	Non-government (Catholic School)	Government (Public School)	Government (Public School)
** Geographic location**^**a**^	Outer Regional	Inner Regional	Outer Regional	Major City
** Total Students enrolled at school**^**a**^	66	162	38	333
** School M:F (%)**^**a**^	57:43	54:46	66:34	56:44
** School ICSEA**^**ab**^	967	1026	929	976
** School ICSEA Percentile (%)**^**ac**^	31	59	18	36
** Bottom ICSEA Distribution (%)**^**ad**^	46	5	53	37
** Language other than English (%)**^**a**^	7	6	23	19
** Indigenous students (%)**^**a**^	9	7	24	13
** Food-related programs at school**	School Garden, Chickens	School Garden	School Garden	School Garden
**Teacher characteristics**				
** FEAST Implemented**	Term 4, 2020	Term 2, 2021	Term 2, 2021	Term 2, 2021
** No. of Teachers Involved**	1	1	1	2
** Gender**	F	F	F	M & F
**Student characteristics**				
** No. of Students Interviewed**	5	7	6	8
** M:F (n)**	2:3	5:2	4:2	4:4
** Age (Mean** ± **SD)**	11.2 (0.84)	11.3 (0.45)	10.8 (0.84)	11.4 (0.55)
** Age Range (years)**	10–12	10–12	10–12	10–12
** Grade: Yr 4: Yr 5: Yr 5|6: Yr 6 (n)**	0:2:1:2	0:4:0:3	2:2:0:2	0:4:0:4
** No. of Focus Groups**	2	2	1	2
** Ethnicity**	*n* = 5 Anglo-Saxon	*n* = 6 Anglo-Saxon	*n* = 5 Anglo-Saxon	*n* = 5 Anglo-Saxon
		*n* = 1 Italian	*n* = 1 Filipino	*n* = 1 Filipino
				*n* = 1 Indigenous
				*n* = 1 Torres Strait Islander

^a^Information from myschool.edu.au (Education Department government website) [62]

^b^ICSEA Index of Community Socio-Educational Advantage values range from 500–1300 representing schools from extremely disadvantaged to advantaged backgrounds (respectively) (mean of 1000 ± 100) [62]

^c^ICSEA percentile e.g. 31%—means that this school is more educationally advantaged than 31% of schools in Australia

^d^Bottom ICSEA Distribution (%) e.g. 46th percentile—means 46% of students in that school are in the bottom quarter of disadvantage, relative to other students; Yr 5|6 is a composite class including students from Grades 5 and 6 in the same classroom; *M* Males, *F* Females, No. Numbers

Four major themes emerged from the analysis of children’s perspectives of how to inspire their peers and families to eat more vegetables in the home and school settings: (i) taste; (ii) family environment; (iii) healthy eating; and (iv) change makers, and one minor theme: sequence of cooking F&V recipes. Figure 2 presents a summary of findings, showing taste, family environment and healthy eating awareness, served as both facilitators and barriers.

### Major theme 1: Taste

‘Taste’ was a salient theme and comprised of three subthemes with two barriers: ‘don’t like taste/vegetables’ and ‘prefer other tastes’ and one facilitator: ‘masking vegetables’.

#### Don’t like taste/vegetables

The subtheme, ‘don’t like taste/vegetables’ was a barrier to children consuming vegetables themselves and implied or stated as a barrier for others not consuming vegetables. The children expressed largely sensory reasons for a dislike of vegetables, such as:

“So pretty much it's like the look, the taste and the feeling.” (School 4, FG 2, Girl 1, 10 yrs, Yr 4).

Dislike for vegetables was predominantly described in terms of not liking the taste, in particular, not liking the taste of vegetables on their own:

“… if you eat them straight, they don’t really taste nice.” (School 2, FG 2, Boy 1, 12 yrs, Yr 6).

#### Prefer other tastes

The most common barrier reported by this group of children, was that they and other children preferred other tastes to the taste of vegetables. More specifically the ‘other tastes’ fell into two categories of processed and discretionary foods such as: sugar/sugary treats/lollies:

“I think it's the fact that you've tasted other foods and you prefer them. Like if you have sugary foods then, if you have the option of … a Nutella sandwich or a pile of carrots, you take a Nutella sandwich.” (School 2, FG 2, Boy 1, 12 yrs, Yr 6)

or fast foods/junk foods/take away foods: examples included McDonald’s, Happy Meals, KFC, Zinger boxes, fatty tastes and salty nuggets:

“Because I want… take away and Zinger boxes and Happy Meals.” (School 3, FG 1, Boy 5, 12 yrs, Yr 6).

#### Masking vegetables

The facilitator within the theme of ‘taste’, was ‘masking vegetables’. Strategies such as adding other flavours to disliked vegetables was the most popular response to questions like *“What do you think would make it easier for children to eat more vegetables?”* Masking vegetables included: adding, blending, mixing, combining, with/to vegetables, other flavours or ingredients to make vegetables more palatable/tasty, such as, vegetables they liked, sauces, dressings, spices, herbs, and meats:

“… in the FEAST program when we made one of the recipes it had a lot of things that I liked, but then again it … had a lot of things that I didn't like, and mixing the flavour from the two, really helped me to like the ones that I didn't.” (School 2, FG 1, Boy 1, 11 yrs, Yr 5).

Masking vegetables also included ‘hiding vegetables’ i.e. don’t tell children, and cut vegetables up finely and add them to foods they like:

“… hide it [vegetables] in the food … they like … they’ll eat it and they won’t even know they’ve eaten a whole lot of veggies.” (School 3, FG 1, Boy 1, 10 yrs, Yr 4).

### Major theme 2: Family environment

The ‘family environment’ theme comprised two subthemes, one of which was a barrier: ‘not introducing children to vegetables’ and the other a facilitator: ‘cooking vegetables in the home’.

#### Not introducing children to vegetables

Although this barrier, ‘not introducing children to vegetables’ was a minor subtheme, it was important to include as this helps provide one reason why children do not eat vegetables and why the family environment is important to act as a facilitator for children to be introduced to vegetables:

“… also, the parents have never given their children the taste [of]… vegetables … so … that continues down their family, and then the next [generation] … that's … why … the family might just not have vegetables.” (School 2, FG 1, Boy 2, 11 yrs, Yr 5).

#### Cooking vegetables in the home

The facilitator to this theme was: ‘cooking vegetables in the home’*.* The children described what their parents cooked at home for the family and how they added vegetables to family meals, such as omelettes, spaghetti bolognaise, Shepperd’s pie, stir-fries and salads. They also described what their parents added to vegetables to make them taste good, such as sauces, dressings, spices, herbs and meats:

“My dad makes us … pork noodle… but it's got all these different types of vegetables …[he] creates … some spice … I love noodles and … the sauces that he puts … in makes [it] all blend together and it tastes really nice.” (School 2, FG 1, Boy 2, 11 yrs, Yr 5).

The children even described how their mothers often hid vegetables in foods they liked and served it to them, their siblings, cousins or fathers, without telling them that there were vegetables in the meals they prepared:

“One time me and my mum made sausage rolls … mum sneaked carrot … and zucchini and corn, peas, put [them] in the sausage roll.” (School 4, FG 2, Girl 4, 11 yrs, Yr 5).

### Major theme 3: Healthy eating

The ‘healthy eating’ theme comprised two subthemes, one of which was a barrier: ‘unhealthy choices’; and the other a facilitator: ‘equating vegetables to healthy eating’.

#### Unhealthy choices

This barrier, ‘unhealthy choices’, overlapped with the subtheme ‘prefer other tastes’ (from the theme ‘taste’). Children expressed that they, and other children, preferred processed and discretionary foods, such as sugar/sugary treats/lollies, and expressed this in terms of being addicted:

“… they get sugar, they get addicted and then they won't eat their vegetables… and … that's pretty hard if you're addicted to junk.” (School 1, FG 2, Boy 1, 11 yrs, Yr 5/6)

or junk food/take away/fast foods, fatty/salty tastes and discussed this in terms of ‘unhealthy options’:

“When people think of vegetables, they think of it as something really bad like peas and Brussels sprouts and how they never want to eat them, then they just choose … more unhealthy options.” (School 2, FG 1, Boy 2, 11 yrs, Yr 5).

#### Equating vegetables to healthy eating

The facilitator to this theme was: ‘equating vegetables to healthy eating’. Several children suggested that a way to inspire their friends/peers to eat more vegetables, was to actually ‘tell them’ the benefits of eating vegetables:

“Maybe just keep telling them [your friends] that it's [vegetables] good for you and you’ll be very healthy.” (School 4, FG 1, Girl 3, 11 yrs, Yr 6).

They also suggested that others, such as experts, idols, athletes, or the ‘Cool Foods’ people should address the school and tell the students the benefits of eating vegetables:

“We could get someone who knows all about vegetables and can get them to go to talk to people at school.” (School 1, FG 2, Boy 2, 11 yrs, Year 5).

One child shared the perspective that it was best to introduce children to vegetables early, to establish healthy habits:

“To get the kids to eat healthy so often … you get it [vegetables] into them … you get them into the habit of it, while they're young, it will stick with them for the rest of their life.” (School 1, FG 2, Boy 2, 11 yrs, Year 5).

They also equated the recipes from the FEAST program as being healthy recipes because they all included either vegetables or fruits in them:

“… with school lunches, I need more foods that are healthy … other than … junk food, so with the … [FEAST] cooking we've made lots of little snacks, I've said to my parents and my parents have been making them and putting them in my lunch.” (School 4, FG 2, Girl 1, 10 yrs, Yr 4).

When asked what their favourite part of FEAST was, one example was:

*“… making healthy meals and then putting them into a cookbook”* [FEAST class cookbook activity]. (School 3, FG 1, Boy 1, 10 yrs, Yr 4).

### Major theme 4: Change makers

‘Change makers’ includes four subthemes that were generated from the analysis: ‘influencing peers’; ‘influencing school’; ‘influencing family/home’; and ‘influencing FEAST program’. They included: facilitators, role modelling and strategies to inspire others to eat vegetables and reciprocal determinism.

#### Influencing peers

The children had many ideas as to how they could influence other children to eat more vegetables. A common strategy was to cook for, or with them. For example, cooking with friends was popular:

“Say if you’re having a sleep over or something … see if they [friends] want to help cook something … cooking is fun … if you make it fun. I'd probably recommend [vegetable] stir fry.” (School 1, FG 2, Girl 3, 11 yrs, Year 5).

Also, cooking for younger peers at school was another strategy:

“We can go and buddy up with the little kids, and … cook recipes and tips … help them cook … teach them about vegetables …” (School 1, FG 2, Girl 3, 11 yrs, Year 5).

Role modelling for peers, by eating vegetables in front of them, and bringing vegetables to school and sharing them was another common strategy:

“Eating them [vegetables] myself right in front of them [friends], or if they’re crunchy, sit next them just go …” [imitates chewing on invisible vegetable and makes sound of crunching]. (School 1, FG 2, Boy 2, 11 yrs, Year 5).

When it came to influencing their friends:

“… when you're best friends, most likely you and your best friend often will be almost the …exact same … if you eat that food, they want to eat that food so, if you want to eat vegetables, they want to eat vegetables.” (School 2, FG 2, Boy 1, 12 yrs, Yr 6).

Another strategy to influence their friends was getting others (for example, idols and athletes) to be the source of inspiration and to role model eating vegetables:

“If they [children] … see … their role models or idols eating like a veggie … just like vegetables … what they would probably think of, she's so cool doing that, or he [is] so cool doing that, let me try …” (School 2, FG 2, Girl 3, 12 yrs, Yr 6).

There were also some suggestions about using imaginary or book characters, to influence younger children:

“If it’s a big and strong character, the child’s favourite character, if a child doesn’t … want to eat their vegetables, tell them the character eats them and is big and strong, it’s very healthy.” (School 4, FG 1, Girl 3, 11 yrs, Yr 6).

#### Influencing school

The children also had many ideas as to how they could influence their school. Some common strategies were to: cook for the school; promote vegetables with handouts and posters; play ‘vegetable’ games; do fundraisers and to promote the FEAST program to other classes within their schools or to other schools within their district or state:

“We could make … fundraisers [at school] and raise money for OzHarvest, and then cook the [FEAST] recipes.” (School 2, FG 1, Girl 3, 11 yrs, Yr 5).

In particular, the canteen was a popular target for them to influence their school with suggestions such as: cook for the canteen; change the canteen food; add free vegetables to the canteen:

“Promote it … send it … to parents … that they're going to be changing it [canteen food] to something healthier that could be better for them…. to try and influence their children and maybe use … [FEAST] recipes to show them how good it is.” (School 2, FG 1, Boy 1, 11 yrs, Yr 5).

#### Influencing family/home

The children also had many ideas as to how they could influence their families and home. Cooking came up again as a popular strategy, to cook with, or for their family:

“… maybe you can ask your family if you can cook with them and give ideas, maybe … tell them what you’ve learnt at school …” (School 4, FG 1, Girl 3, 11 yrs, Yr 6).

#### Influencing FEAST program

When the children were asked *“How would you improve the FEAST program?”* the common theme that arose was they wanted ‘more’. More time in the cooking classes and more terms in the school year to enjoy FEAST; *‘more recipes’* (e.g. breakfast, dinner and dessert recipes); *‘more challenging recipes’*; more variety in ingredients (e.g. meat, other vegetables); more healthy food recipes; more choices; more cooking and cooking styles (i.e. baking, roasting); and more recipes to *‘reuse food before it goes to waste’*.

Of note, was that when asked *“what was your favourite part of FEAST”*, the most common response was cooking, followed by cooking with their peers.

“… more time, overall, over all the [school] terms and kind of more time during class to create the recipes, so maybe you can do [cook] … more than one [recipe] in each session.” (School 4, FG 2, Girl 2, 11 yrs, Yr 5).

### Minor theme: Sequence of cooking F&V recipes

A minor theme that arose ‘sequence of cooking F&V recipes’, could be a potential facilitator and was identified by one participant as a means to increase vegetable consumption. When asked what made him start eating vegetables during the FEAST program he replied:

“we [made]mostly all the ones that didn't have vegetables… at the start of FEAST program.” (School 1, FG 2, Boy 1, 11 years, Yr 5/6),

and one of the other participants added:

“and by the end we're eating … a lot of vegetables.” (School 1, FG 2, Girl 3, 10 years, Yr 5).

and he then concurred:

*“a lot of vegetables, yes!”* (School 1, FG 2, Boy 1, 11 years, Yr 5/6).

We can infer that the teacher started their class cooking activities with the non-vegetable-based recipes at the beginning of the FEAST program and progressed to the vegetable-based recipes.

## Discussion

The KiiDSAY project aimed to explore children’s perspectives of how they would inspire vegetable consumption among their peers and families, in the home and school environments, after they had participated in the FEAST program, as well as gaining insights into how the program could be enhanced. Four major themes were generated from the data analysis: ‘taste’; ‘family environment’; ‘healthy eating’; and ‘change makers’, with one minor theme, ‘sequence of cooking F&V recipes’. Overall, the children wanted to improve the taste of vegetables and inspire peers and families by using cooking as a means of influencing others to eat more vegetables at home and school. They wanted to be good role models or find good role models to highlight the benefits of vegetables to their peers. They also wanted to change their school environment (i.e. canteen) to support vegetable consumption. As they enjoyed cooking activities with their peers the most, they wanted to enhance the FEAST program by adding more cooking, more recipes, more time, and introducing the program to younger peers in their school and to other schools. This desire to do more cooking and use cooking as a means of influencing others to eat more vegetables is consistent with children’s need to upskill their 'food literacy' skills, where 'food literacy' includes both knowledge and skills acquisition, in relationship to food and healthy eating [62].

The theoretical frameworks that were used to develop the interview focus group questions were also used to analyse data. Reviews published on school-based nutrition programs, reporting the use of behaviour change theories like SCT and EMHB, report findings that support the notion that those programs are more likely to promote healthy eating (including the increased consumption of F&Vs) among children. The children’s responses in this study produced answers that were consistent with behaviours that would support the increased consumption of vegetables (i.e. cooking with peers and family, improving taste of vegetables, role modelling, and desiring changes in school environment to support the consumption of vegetables).

Collectively, barriers to eating vegetables raised by the KiiDSAY participants were consistent with previously published studies that have reported children do not like the taste of vegetables—not in Australia [63], nor in other countries around the world [22, 32, 35, 37, 38]. Furthermore, children prefer other tastes, that of ‘unhealthy foods’ or discretionary foods [36, 63, 64] because they ‘taste better’ [31]; are easier to eat than F&Vs [39, 63]; and easy to access in the home and school environments [37, 63, 65]. Also, consistent with the literature [66–68], KiiDSAY participants suggested that not being exposed to vegetables in the home creates a barrier for children’s uptake, liking and consumption. Collectively, facilitators to eating vegetables suggested by the KiiDSAY participants were also consistent with published research, such as making them more palatable/tasty [22, 37, 38], adding them to foods they like [35, 69], or combing them with other foods [38, 39] i.e. adding condiments or hiding them.

To overcome the major barrier of taste, and to facilitate enhancing the flavour of vegetables, one strategy stood out, and that was cooking. Cooking was consistently raised and underpinned all suggestions to influence peers and families to consume more vegetables, confirming current practice and knowledge regarding the nutritional benefits of cooking activities [19, 70–72]. Involving children in food preparation and cooking activities has produced increases in vegetable consumption in a variety of settings, including culinary cooking centres [73]; farming camps [74]; cooking workshops [75]; and schools [76–82]. Children have reported and displayed a natural curiosity to taste vegetable dishes they create and describe feelings of pride related to preparing meals themselves [73, 74]. This notion supports our findings that the KiiDSAY participants suggested cooking for, or with peers, friends, siblings or family, as a way to influence vegetable intake, suggesting that if children were involved in cooking it, they were more likely to try it. Consistent with findings from other qualitative studies [76–82], and systematic reviews [19, 70–72, 83–85], our findings suggest that cooking is a powerful strategy to increase children’s vegetable consumption in school-based nutrition programs.

The flow-on benefits and positive effects that go beyond the school and into the home have been reported from primary-school-based programs such as *the Stephanie Alexander Kitchen Garden* program in Australia [86], *Jamie Oliver's Kitchen Garden Project (JOKGP)* in the UK [87] and the *Delicious and Nutritious Garden*, in the USA [88]. In a qualitative study about the JOKGP*,* both children and adults confirmed that as a result of the program, the children were taking recipes home, and trying them out on their own or with their families [87]. This was a similar finding to our study, where the KiiDSAY participants expressed how they were, or would, influence their families to try new vegetables and new vegetable recipes in the home. Like these studies [86–88], our findings help to highlight the influence that children can have on food, particularly on vegetable consumption, and cooking in the home.

Studies have reported that peers and friends are influential over other children's dietary behaviours [89, 90]. Positive peer influences can present as peer modelling the consumption of healthy snacks [91]; peer approval [92]; peer concern [93]; and encouragement to eat healthy foods [94]. In a US study (*n* = 2043, mean age = 14.4 ± 2 years), results revealed that adolescents exhibited similarities in healthy eating habits with their best friends and vegetable intakes were significantly related to this relationship [95]. These findings are consistent with suggestions made by the KiiDSAY participants, who wanted to be positive role models for their friends and peers by encouraging them to eat healthily, role model eating salads and vegetables, and sharing and/or cooking with or for them, the FEAST recipes that made vegetables more palatable. These ‘influencer’ effects are particularly promising as school-based experiential programs that include cooking activities, such as FEAST, aspire to have an impact beyond the school level. The mission of OzHarvest’s FEAST program is: *“Inspiring kids to eat healthy, waste less, and be change-makers in the local community”* [96]. Similarly, children as change makers has been reported in other similar school-based nutrition and cooking programs [87, 88]. The ‘change makers’ theme bodes well with OzHarvest, the FEAST program, and with the aims of this study.

Given that increasing vegetable consumption continues to remain challenging, it may be necessary to consider more pragmatic and innovative ways to boost children’s vegetable intakes [97]. One minor theme that was raised during this study, and appears to be unexplored in the literature, is the strategy of sequencing the delivery of F&V recipes. One suggestion would be to change the order of introducing recipes to children during cooking activities, by starting with their most liked tastes (i.e. sweets and fruits) and progressing to their least liked tastes of vegetables [26, 31, 32]. Given children are more likely to taste what they have cooked [73, 74], this could provide greater impetus for them to try recipes that include ingredients they do not like, such as vegetables [26, 31, 32]. Because culinary interventions that include cooking and tasting are known to improve healthy dietary intake [98–100] and self-efficacy [98] among school-aged children, ordering F&V recipes in a particular sequence could well be a pragmatic strategy to investigate in future research studies of this nature.

### Strengths and limitations

This study targeted a cohort who rarely participate in research about their lives [101–103]. By asking children to describe their experiences, views and thoughts, program developers have an opportunity to design improved programs to better cater to children’s needs [36, 104]. The focus group-style interview can help create a safe peer environment [53], allowing the ‘student voice’ to enhance the evaluation of school-based programs [105]. This has the potential to make positive and significant contributions to research [106] and to the FEAST program. Furthermore, to our knowledge, this was the first qualitative study to explore how children would motivate their families to eat more vegetables. Vital to the rigor of a qualitative study, is the carefully described framework for data analysis [46] (detailed in the methods section), as well as credibility, dependability, confirmability and transferability [46, 107, 108] (which are detailed in Additional file 3) along with reflexivity.

A limitation of the study is that it is possible that other perspectives of children’s views could have been captured using a larger sample. Recruitment of schools during Term 4 (last term of the school year) coincided with COVID-19 public health measures in the state of NSW, enforcing school closures for the first half of that school term. The concept of “*data saturation*”, which has become an accepted standard to determine sample size [46], could not be used due to the challenges of recruiting schools during pandemic conditions. Despite these challenges, we are confident that “*sufficient*” [109] findings were elicited from this cohort, due to the richness of data received, with no new patterns arising [110] by the end of the last focus group interview.

Furthermore, the findings of this study may not be transferable to other schools. Even though participants included both boys and girls, as well as schools with an ICSEA within the national average (1000 ± 100) [62], (i.e. with participating schools in the range of 929–1026), however, there were more regional schools (i.e. 3/4, 75%) participating in this study than city schools. Another limitation is that participants were recruited from schools that had been implementing a program that incorporated nutrition and environmental sustainability components (i.e. FEAST), which would not be representative of children from schools that had not participated in such programs.

### Implications for research and practice

Two main elements appear as constants within qualitative description studies in health care research: learning from the participants and their descriptions and using this knowledge to influence future programs [46]. Children’s inputs have the potential to inform future intervention studies involving them [46, 111, 112] and should be considered when designing or refining programs that promote healthy eating [113], including OzHarvest’s FEAST program. This study has several implications for research and practice of the FEAST program and other similar initiatives promoting vegetable consumption among children. Researchers might consider the following points that could potentially improve nutrition school-based programs promoting vegetable consumption by:

- ▪ providing more opportunities for children to cook with their peers and families in the home and school environments;
- ▪ supplying recipes that can ‘hide’ vegetables in foods children like, for instance, grate vegetables and make savoury ‘pancakes’ or vegetable fritters;
- ▪ sequencing the delivery of recipes, i.e. start cooking activities with non-vegetable-based recipes (fruit-based recipes) and progress to vegetable-based recipes;
- ▪ involve role models during cooking activities from the school or community (i.e. older peers, sports people, experts).

Implementers may also consider the following points that could potentially improve children’s vegetable consumption:

- including substantial cooking components into nutrition-based programs;
- running programs that involve cooking activities over longer durations; and
- providing supportive environments within schools to support nutrition-based programs that encourage vegetable consumption.

## Conclusions

This study contributes to the limited knowledge about children’s perspectives of how they would inspire the consumption of vegetables amongst their microsystem (peers and family, in the school and home). Children’s inputs have the potential to inform future interventions and should be taken into consideration when designing or refining school-based nutrition programs, like FEAST, by providing more opportunities for children to cook with their peers and families in the home and school environments. This would entail supplying recipes that hide/mask/enhance the flavour of vegetables, sequencing the delivery of recipes from fruit-based through to vegetable-based recipes and involving role models (peers and community) during cooking activities. Given the challenges faced in increasing children’s vegetable consumption, particular focus on this area is warranted.

### Supplementary Information

**Supplementary Material 1.**

**Supplementary Material 2.**

**Supplementary Material 3.**

## Data Availability

The datasets created and analyzed during the present study are available from the corresponding author upon reasonable request, subject to ethical approval.

## Abbreviations

KiiDSAY: Kids initiative inspires Dietary Success in Adults and Youth
FEAST: Food Education and Sustainability Training
F&V: Fruit and Vegetable
QD: Qualitative description
COREQ: The Consolidated Criteria for Reporting Qualitative Research
PPM: PRECEDE-PROCEED Planning Model
SCT: Social Cognitive Theory
EMHB: Ecological Model of Health Behaviour
FG: Focus group
NSW: New South Wales
ICSEA: Index of Community Socio-Educational Advantage
JOKGP: Jamie Oliver's Kitchen Garden Project
