# Supplementary material for: Primary-school-aged children inspire their peers and families to eat more vegetables in the KiiDSAY project: a qualitative descriptive study
Source: BMC Pediatr. 2024 Mar 9;24:175. doi: 10.1186/s12887-024-04643-z (PMC10924354; doi:10.1186/s12887-024-04643-z)
Supplement: Supplementary file 1 — Supplementary Material 1. [file 12887_2024_4643_MOESM1_ESM.pdf]

Additional file 1: Focus Group Interview Questions, Key Constructs and Theoretical Frameworks for KiiDSAY Project

	Focus group interview questions	Theoretical Frameworks and Key Constructs	Definitions of key constructs
**A. FEAST Feedback Questions**	Q1. What did you like the most about FEAST?<br>(Prompt: what did you think of the cooking classes?)<br>Q2. If you could, how would you make FEAST better?	Experience – Phenonomenolgy<br>SCT [1] and EMHB [2] - Reciprocal Determinism	Reciprocal Determinism - behaviour is influenced bidirectionally, between the individual and their environment; children are influenced by and can influence their environment (microsystem i.e. peers, family, home, school)
**B. Vegetable Intake Questions**	Q3. Do you think that FEAST helped you to eat more vegetables?<br>Q3a. If 'yes', how? Or… If 'no', why not?<br>Prompt: What do you think will help you to eat more vegetables?<br>Q4. What do you think prevents you from eating vegetables?<br>Q5. What do you think would make it easier for children to eat more vegetables?	SCT - Behaviour change<br>SCT - Facilitators<br>SCT - Barriers<br>SCT - Facilitators	Behavioural capacity – to be capable of change, which depends on knowledge and skills.<br>Facilitator- perceived facilitators to behavior change.<br>Barriers- perceived Barriers to behavior change<br>Facilitator- perceived facilitators to behavior change
**C. Influencer Questions**	Q6. Do you think you could help your friends to eat more vegetables?<br>(Prompt: inspire, influence, motivate, encourage)<br>Q6a. If 'yes', how? Or… If 'no', why not?	SCT - Reciprocal Determinism and Role Models<br>EMHB - Reciprocal Determinism	Reciprocal Determinism- behaviour is influenced bidirectionally, between the individual and their environment.<br>Role models- the behaviors of people that individuals copy.

	Q7. Do you think you could help your family to eat more vegetables?<br>(Prompt: inspire, influence, motivate, encourage)<br>Q7a. If 'yes', how? Or… If 'no', why not?	SCT - Reciprocal Determinism and Role Models<br>EMHB - Reciprocal Determinism	AA
**D. Actions**	Q8a. What actions would you take to share *your* ideas with your class/school?<br>Q8b. What actions would you take to share *your* ideas with your family?	SCT - Reciprocal Determinism<br>EMHB - Reciprocal Determinism<br>SCT – Strategies	Reciprocal Determinism - Create and change their environments; be the change makers among their peers/family/school<br>Strategies - or plans to realize goals or actions

Legend: *FEAST* Food Education and Sustainability Training; *SCT* Social Cognitive Theory [1]; *EMHB* The Ecological Model of Health Behaviour [2]

References:

1. Bandura A: Health Promotion by Social Cognitive Means. *Health Educ Behav* 2004, 31(2):143-164.
2. Bronfenbrenner U: Ecological models of human development. In: *International Encyclopedia of Education. Volume Vol. 3*, 2nd edn. Edited by Husen T, Postlethwaite TN. Oxford, England: Pergamon Press/Elsevier Science; 1994: 1643-1647.
