# Supplementary material for: Primary-school-aged children inspire their peers and families to eat more vegetables in the KiiDSAY project: a qualitative descriptive study
Source: BMC Pediatr. 2024 Mar 9;24:175. doi: 10.1186/s12887-024-04643-z (PMC10924354; doi:10.1186/s12887-024-04643-z)
Supplement: Supplementary file 2 — Supplementary Material 2. [file 12887_2024_4643_MOESM2_ESM.pdf]

INTERVIEW GUIDE- KiiDSAY Project

For students in Grades 5 & 6 (aged 10-12 years)
To be conducted during lunch break. Lunch breaks in NSW primary schools are one hour long.
This interview should take between 40-45 minutes.
The class teacher or another school staff member must be present to supervise this group interview.
To be conducted on the Zoom platform using the teachers computer.

Project Title:
KiiDSAY (KIDS initiative inspires DIETARY SUCCESS in ADULTS and YOUTH) project: qualitative description study.

Research Question:

How can school-aged children inspire their peers and families to eat more vegetables in the home and school environments?

Pre-Introduction:
Before we begin, I'd like to inform you that this interview will be recorded.

START RECORDING

Introduction:

Firstly, my name is Fay, and I'm comfortable with you all calling me Fay during today's discussion. I'd like to thank you for agreeing to participate in the KiiDSAY project. Thank you for providing signed consent forms from your parents. I would like to ask you now for your verbal consent, in order to start this interview. So, if you are all happy to participate you can say "Yes, I consent to being interviewed and my answers recorded".
Thank You.

I'm looking forward to asking you about what you thought of FEAST and how you would go about helping your friends, classmates, your school and family to eat more vegetables.

Your teacher chose you for this project because they believe you have something valuable to contribute to this project. It's important today that we practice listening attentively and respecting each other's ideas and opinions. There are no 'right' or 'wrong' answers, only your ideas and opinions, and they are all important to this project. It would be lovely to hear from all of you today. You and I are collaborators in this research project, and together we have a problem solve.

I'd also like you to know that if any question/s make you feel uncomfortable, you have the right to choose not to answer, you can just say 'pass' or you can withdraw from the interview. You can do that without any consequences and without giving any reason.

Let's start by introducing ourselves and saying something about what we like to do, for example, I like reading, playing sport, singing and then, name your favourite fruit and vegetable, please. I will start, to give you an example. My name is Fay and I enjoy painting. I like mangoes and sweet potatoes. Who would like to go next?

A: FEAST Feedback Questions:
Q1. What did you like the most about FEAST?
(Prompt: what did you think of the cooking classes?)

Q2. If you could, how would you make FEAST better?

B: Vegetable Intake Questions:

Q3. Do you think that FEAST helped you to eat more vegetables?
Q3a. If 'yes', how? Or… If 'no', why not?
Prompt: What do you think will help you to eat more vegetables?
Q4. What do you think prevents you from eating vegetables?
Q5. What do you think would make it easier for children to eat more vegetables?

C: Influencer Questions:

Q6. Do you think you could help your friends to eat more vegetables?
(Prompt: inspire, influence, motivate, encourage)
Q6a. If 'yes', how? Or… If 'no', why not?
Q7. Do you think you could help your family to eat more vegetables?
(Prompt: inspire, influence, motivate, encourage)
Q7a. If 'yes', how? Or… If 'no', why not?

D: Actions

Q8a. What actions would you take to share *your* ideas with your class/school?
Q8b. What actions would you take to share *your* ideas with your family?

Closing

Thank you so much for participating in this project, by allowing me to interview you.
Your answers are very valuable to this research project.

At this point I'd like to let you know that if you choose to withdraw your interview from the KiiDSAY project, you are free to do so at any time, up until one month from today, without any consequences.

Your identity will be protected and will not be used at any time. The recording of your voice will be stored securely as explained in the plain language statement, and will be destroyed five years after the project is published.

Your teacher will receive the results of the entire KiiDSAY project, so please feel free to ask them to share the results with you. With your teacher's permission, you will be able to discuss a plan of action so that you can share the results with your class and school community.

If there is anything else, your teacher can contact me via email. My details were listed on the plain language statement.

Thank You

END RECORDING
