# Supplementary material for: Primary-school-aged children inspire their peers and families to eat more vegetables in the KiiDSAY project: a qualitative descriptive study
Source: BMC Pediatr. 2024 Mar 9;24:175. doi: 10.1186/s12887-024-04643-z (PMC10924354; doi:10.1186/s12887-024-04643-z)
Supplement: Supplementary file 3 — Supplementary Material 3. [file 12887_2024_4643_MOESM3_ESM.docx]

**Additional file 3**: Strategies to assess rigor in qualitative research and its application to the KiiDSAY project

| **Rigor criteria** | **Purpose** | **Original strategies** | **Strategies applied in KiiDSAY project to achieve rigor** |
| --- | --- | --- | --- |
| **1. Credibility**  (Internal validity) | To establish confidence that the results (from the perspective of the participants) are true, credible and believable. | Prolonged and varied engagement with each setting | Prolonged engagement was not possible due to COVID19 school closures during the school term the interviews were conducted.  However, interviewer established rapport with participants before commencing focus group interviews. Interviewer shared her hobby (art/painting) and her favourite fruit (mango) and vegetable (sweet potato) with the participants, and then asked them to do the same. They all shared with interviewer. |
|  |  | Interviewing process and techniques | Interview protocol pilot tested with one 12-year-old girl.  Interview techniques used to improve the focus group discussions from Adler et al 2019^1^:  Children were not more than ±2 years age difference within the group; on average 4-8 children participated per focus group; questions were short, concrete, specific, and easily understandable questions, no acronyms, jargon, or technical language was used; 8- to 12-year olds were not interviewed for more than 60-90 minutes; “assent” as a verbal or nonverbal confirmation – was asked for prior to commencement even though parents/carers had provided written consent; interviewer introduced themselves with their first name and accepted being addressed informally (to help children perceive their relationship with the interviewer as more informal than their relationship with their teachers); used icebreakers to make children feel relaxed and to make it easier for children to start the discussion (interviewer introduced herself using her first name and shared her favourite fruit, vegetable and hobby and asked the children to share the same about themselves) |
|  |  | Collection of referential adequacy materials | Notes taken during and after interviews as well as during an analysis. Notes discussed with RL and AW (co-authors). |
|  |  | Establishing investigators’ authority | Interviewer had training, previous experience in interviewing, knowledge and research skills to perform their role. |
|  |  | Peer debriefing | Discussed focus group interviews with co-authors following the first three interview sessions. |
| **2. Dependability**  (Replicability) | To ensure the findings of this qualitative inquiry are repeatable if the inquiry occurred within the same cohort of participants, coders and context. | Rich description of the study methods | Detailed drafts of the study protocol were prepared prior to this study, discuss with co-authors, and enhanced as needed throughout the study. |
|  |  | Establishing an audit trail | Notes taken during and after interviews also during analysis. |
|  |  | Stepwise replication of the data | Two members of the research team who were experts in qualitative research independently cross-checked primary authors coding and emerging themes and subthemes for 4 focus group interviews.  All co-authors discussed and agreed on the final themes and subthemes. |
| **3. Confirmability**  (Objectivity) | To extend the confidence that the results would be confirmed or corroborated by other researchers. | Reflexivity | Reflexivity – interviewer’s core values: family, health and education; is a retired health professional; an author of evidence-based children’s book series; educator of patients, public and professionals; health promotion of healthy lifestyle activities; of Greek heritage; cooks the Greek Mediterranean cuisine a healthy and sustainable way to eat; cooks for family, friends and for workshops.  Interviewer’s experience with interviewing children: as a retired health professional interviewer has had six years of experience interviewing children in clinical settings; in 2013 interviewer conducted her three focus group interviews to garner information relating to the children’s books she was going to publish, amongst boys and girls, aged 5-12 years, with focus group sizes ranging between 8-12 children.  Interviewer has only conducted one previous qualitative research project involving adults (one-on-one interviews via Zoom platform). |
|  |  | Triangulation  (Theoretical; Investigator; Data Source; Methodological) | Theoretical Triangulation - codes were embedded within key constructs of the Social Cognitive Theory (barriers, facilitators, role models, strategies and reciprocal determinism) and within the Ecological Model of Health Behaviour (reciprocal determinism) |
|  |  |  | Investigators Triangulation - Two members of the research team cross checked coding and emerging themes and subthemes. RL is a  Postdoctoral Research Fellow in public health nutrition and food security. She has experience in qualitative research including thematic analysis and interviews. AW is an Accredited Practising Dietitian and National Course Coordinator of the Master of Dietetic Practice with extensive experience in qualitative research. |
|  |  |  | Data Source Triangulation – interviewer repeated back to participants the answers and asked them to confirm that the interviewer had understood their responses and perspectives, after each response.  However, we could not include member check procedures, due to COVID19 closures to schools. Disruptions to this study’s timeline, meant that this study was conducted within the last two weeks of the 2021 scholastic year and schools were closing for six weeks for summer holidays. As a result no follow-up member checks were possible. |
|  |  |  | Methodological Triangulation – Data from this qualitative study triangulated with the feedback from the FEAST survey open-ended questions embedded in a quantitative survey used during the FEAST pilot, as well as the FEAST impact and process evaluation. Data also triangulated with the results found in other qualitative studies as well as from quantitative studies, including systematic reviews. |
| **4. Transferability**  (External validity) | To extend the confidence that the results would be confirmed or corroborated by other researchers | Purposeful sampling to form a nominated sample | Sampling was purposive (i.e. children who had participated in the FEAST program implementation).  Convenience sampling (i.e. children who had participated in the FEAST program prior to COVID19 school closures)  Maximum variation (boys and girls; rural and city schools; government and non-government schools) |
|  |  | Data saturation | Could not be applied due to the challenges of recruiting schools during pandemic conditions, with school closures occurring during the school terms this study was scheduled for. |

Adapted from Guba and Lincoln^1^ and Forero et al., 2018.^2^

**References**

1. Guba EG, Lincoln YS: Epistemological and methodological bases of naturalistic inquiry. *ECTJ* 1982, 30(4):233-252.

2. Forero R, Nahidi S, De Costa J, Mohsin M, Fitzgerald G, Gibson N, McCarthy S, Aboagye-Sarfo P: Application of four-dimension criteria to assess rigour of qualitative research in emergency medicine.

*BMC Health Serv Res* 2018, 18(1):120.
